# Phase 2 Trial of Enfortumab Vedotin in Patients With Previously Treated Locally Advanced or Metastatic Urothelial Carcinoma in China

**DOI:** 10.1002/cam4.70368

**Published:** 2024-11-12

**Authors:** Siming Li, Yanxia Shi, Haiying Dong, Hongqian Guo, Yu Xie, Zhongquan Sun, Xiaoping Zhang, Eric Kim, Jun Zhang, Yue Li, Chenming Xu, Haishan Kadeerbai, Sue Lee, Seema Gorla, Jun Guo, Xinan Sheng

**Affiliations:** ^1^ Department of Genitourinary Oncology, Key Laboratory of Carcinogenesis and Translational Research (Ministry of Education/Beijing) Peking University Cancer Hospital & Institute Beijing China; ^2^ Department of Medical Oncology, State Key Laboratory of Oncology in South China, Collaborative Innovation Center for Cancer Medicine Sun Yat‐sen University Cancer Center Guangzhou China; ^3^ Department of Urology Zhejiang Provincial People's Hospital Hangzhou China; ^4^ Department of Urology, Drum Tower Hospital Medical School of Nanjing University, Institute of Urology, Nanjing University Nanjing China; ^5^ Department of Urology, Hunan Cancer Hospital and the Affiliated Cancer Hospital of Xiangya School of Medicine Central South University Hunan China; ^6^ Department of Urology Huadong Hospital, Fudan University Shanghai China; ^7^ Department of Hematology and Oncology (Key Department of Jiangsu Medicine) Zhongda Hospital, Southeast University Medical School Nanjing Jiangsu China; ^8^ Pfizer Inc Bothell Washington USA; ^9^ Astellas (China) Investment co., Ltd. Beijing China; ^10^ Astellas Pharma Inc. Northbrook Illinois USA

**Keywords:** antibody–drug conjugates, efficacy, monomethyl auristatin E, safety, urothelial carcinoma

## Abstract

**Background:**

Enfortumab vedotin, a fully human monoclonal antibody–drug conjugate (ADC) directed to Nectin‐4, prolonged overall survival (OS) versus standard chemotherapy in patients with previously treated locally advanced or metastatic urothelial carcinoma (mUC) previously receiving a programmed cell death protein 1/ligand 1 (PD‐1/L1) inhibitor and platinum‐based chemotherapy in the pivotal, phase 3 EV‐301 clinical trial, supporting global approvals of enfortumab vedotin monotherapy. This bridging study was the first to evaluate enfortumab vedotin monotherapy in previously treated Chinese patients with locally advanced or mUC.

**Methods:**

EV‐203 was a multicenter, open‐label, phase 2 study (NCT04995419) assessing efficacy, safety/tolerability, pharmacokinetics (PK), and immunogenicity of enfortumab vedotin in 40 Chinese patients (PK analysis set, *n* = 13) with previously treated locally advanced or mUC. Patients received enfortumab vedotin 1.25 mg/kg (Days 1, 8, and 15). Primary endpoints included confirmed objective response rate (ORR) by the independent review committee (IRC) and PK parameters of ADC, total antibody (TAb), and free monomethyl auristatin E (MMAE). Secondary endpoints included investigator‐assessed confirmed ORR; investigator‐/IRC‐assessed duration of response (DOR), disease control rate (DCR), and progression‐free survival (PFS); OS; immunogenicity; and safety/tolerability.

**Results:**

As of May 13, 2022, the median follow‐up was 6.5 months. Confirmed ORR was 37.5% (n/*N* = 15/40; 95% CI: 22.7%–54.2%) by IRC and 42.5% (n/*N* = 17/40; 95% CI: 27.0%–59.1%) by investigator assessment. By IRC, DCR was 72.5% (*n* = 29), median DOR was not reached, and median PFS was 4.7 months. Median OS was not reached. Endpoints assessed by investigators were consistent with IRC assessments. Two patients discontinued treatment for treatment‐related adverse events. No new safety signals were identified. ADC, TAb, and free MMAE were characterized in Chinese patients and consistent with previously characterized populations. The incidence of positive antitherapeutic antibodies postbaseline was 0%.

**Conclusion:**

Enfortumab vedotin demonstrated meaningful clinical activity with a manageable safety profile in Chinese patients with previously treated locally advanced or mUC.

**Trial Registration:**

ClinicalTrials.gov identifier: NCT04995419

## Graphical abstract

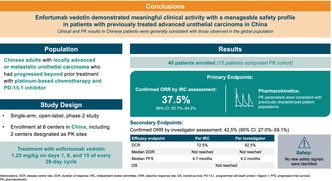

## 1 Introduction

Urothelial carcinoma (UC) comprises carcinomas of the lower (bladder and urethra) and upper (ureter, renal pelvis, renal calyces) urinary tracts [1]. UC that originates in the bladder accounts for more than 90% of all bladder cancer cases [2], and it is the ninth most common cancer worldwide, with more than 610,000 new cases diagnosed in 2022 [3, 4]. In China, over 92,000 new cases of bladder cancer were diagnosed and approximately 41,000 deaths related to bladder cancer were estimated to have occurred in 2022 [5]. Projections through the year 2030 suggest an upward trend in the incidence of bladder cancer in China [6].

According to global treatment guidelines, the preferred first‐line treatment option for locally advanced or metastatic UC (mUC) is enfortumab vedotin and pembrolizumab [7, 8, 9]. Additional treatment options for locally advanced or mUC include nivolumab combined with gemcitabine and cisplatin for cisplatin‐eligible patients, as well as platinum‐based chemotherapy followed by avelumab maintenance therapy [7, 8, 10, 11, 12]. However, the preferred first‐line treatment for locally advanced or mUC in China remains platinum‐based chemotherapy, due to drug availability [13]. As second‐line therapy, programmed cell death protein 1/ligand 1 (PD‐1/L1) inhibitors produce durable responses in a minority of patients [14, 15, 16, 17], while disitamab vedotin, a human epidermal growth factor receptor 2 (HER2)–targeted antibody–drug conjugate (ADC) is limited to patients with HER2 overexpression (IHC2+ or 3+), present in only 13% to 44% of patients with UC [18, 19, 20], who received platinum‐based chemotherapy [13]. Erdafitinib is also recommended for patients with susceptible *FGFR3* genetic alterations after prior use of platinum‐based chemotherapy and an immune checkpoint inhibitor [7, 8, 21]. However, only 21% to 28% of patients with mUC are eligible for erdafitinib based on the presence of tumors that have susceptible *FGFR3* genetic alterations, and the drug is not currently approved in China [22, 23]. Five‐year survival prognosis remains at less than 10% for patients with metastatic disease [24]. As such, a need remains for effective and well‐tolerated treatment options that can improve survival in patients in China with locally advanced or mUC whose disease has progressed despite having received currently approved and available treatments in China, namely platinum‐based chemotherapy and PD‐1/L1 inhibitor therapy [25].

Enfortumab vedotin is a fully human monoclonal ADC directed to Nectin‐4, delivering intracellular monomethyl auristatin E (MMAE; a microtubule‐disrupting agent) to tumor cells resulting in cell cycle arrest and apoptotic cell death [26, 27, 28]. In a single‐arm phase 2 trial in patients with locally advanced or mUC previously treated with platinum‐based chemotherapy and PD‐1/L1 inhibitors, enfortumab vedotin treatment led to an objective response rate (ORR) of 44% and a median duration of response (DOR) of 7.6 months [27]. In the pivotal phase 3 EV‐301 trial, enfortumab vedotin demonstrated superior overall survival (OS) versus standard chemotherapy (median OS, 12.88 vs. 8.97 months, respectively; hazard ratio [HR] 0.70; 95% confidence interval [CI]: 0.56–0.89; *p* = 0.001) in patients with previously treated locally advanced or mUC [29]. Data from the phase 3 trial supported approval of enfortumab vedotin globally for the treatment of adults with locally advanced or mUC who previously received a PD‐1/L1 inhibitor and platinum‐based chemotherapy [28, 30].

EV‐203 (NCT04995419) was a bridging study to the global phase 2 (EV‐201) and phase 3 (EV‐301) studies designed to evaluate the efficacy, safety, immunogenicity, and pharmacokinetics (PK) of enfortumab vedotin in previously treated Chinese patients with locally advanced or mUC. This trial is the first to study enfortumab vedotin as monotherapy for this indication in China.

## 2 Methods

### 2.1 Trial Design and Oversight

This was a single‐arm, open‐label phase 2 study that enrolled patients from six centers in China. Up to 40 patients were planned for enrollment. Two of the study centers were designated as PK cohort sites and planned to enroll 12 or more patients who agreed to intensive PK sampling for PK analysis in addition to safety and efficacy assessments.

The protocol and amendments were approved by independent review boards or ethics committees at each site. The study was conducted in accordance with the Declaration of Helsinki, the Council for International Organizations of Medical Sciences ethical guidelines, and applicable International Council for Harmonisation of Technical Requirements for Pharmaceuticals for Human Use Good Clinical Practice guidelines. All patients (or a legally authorized representative) provided written informed consent.

### 2.2 Patients and Procedures

The study enrolled Chinese adults (age ≥ 18 years or considered an adult per local regulations) with histologically or cytologically confirmed UC/transitional cell carcinoma of the bladder, renal pelvis, ureter, or urethra not amenable to curative intent treatment. Patients with other histologies, including adenocarcinoma, squamous differentiation, or mixed, were also eligible. Patients must have received prior treatment with platinum‐based chemotherapy and PD‐1/L1 inhibitor therapy in the locally advanced or metastatic setting or in the neoadjuvant or adjuvant setting with recurrence or progressive disease during therapy or within 12 months (platinum‐based chemotherapy) or 3 months (PD‐1/L1 inhibitor) of completion of treatment; had baseline measurable disease according to Response Evaluation Criteria in Solid Tumors (RECIST) version 1.1; and an Eastern Cooperative Oncology Group performance status (ECOG PS) of 0–1. Key exclusion criteria were preexisting grade 2 or higher sensory/motor neuropathy; active central nervous system metastases; clinically significant toxicity associated with prior treatment; ongoing grade 3 or higher immunotherapy‐related hypothyroidism or panhypopituitarism, ongoing immunotherapy‐related colitis, uveitis, myocarditis, or pneumonitis; other immunotherapy‐related adverse events (AEs) requiring high‐dose steroids (prednisone > 20 mg/day or equivalent); or history of uncontrolled diabetes mellitus (glycated hemoglobin ≥ 8% or between 7% and < 8% with diabetes‐related symptoms not otherwise explained) within 3 months prior to the first study dose.

Patients were treated with intravenous enfortumab vedotin 1.25 mg/kg (maximum dose, 125 mg) for approximately 30 min on Days 1, 8, and 15 of every 28‐day cycle. Study treatment continued until one or more of the following occurred: patient or investigator decision; documented radiologic disease progression per RECIST version 1.1 by investigator assessment; start of new anticancer therapy; unacceptable toxicity; pregnancy; protocol noncompliance; or more than six consecutive doses of study treatment missed by the patient. Prespecified dose modifications were permitted depending on AE type and severity.

### 2.3 Assessments

Antitumor activity was assessed every 8 weeks (±1 week) via computed tomography with contrast. After 1 year of study, imaging frequency was reduced to every 12 weeks (±1 week). Complete response (CR) and partial response (PR; per RECIST version 1.1) were confirmed by repeat scans conducted at least 4 weeks after the initial response. Responses were assessed by investigators and an independent review committee (IRC). Tumor assessments were continued until the study end or the first occurrence of radiologic disease progression per RECIST version 1.1, as determined by investigator assessment, the patient initiated new anticancer therapy, or the patient died, withdrew study consent, or was lost to follow‐up.

Safety assessments included physical and ophthalmologic examinations, clinical laboratory tests, 12‐lead electrocardiography, and ECOG PS.

In the PK cohort (i.e., PK analysis set), intensive PK sampling was performed wherein blood samples for analysis of ADC, total antibody (TAb), and free (unconjugated) MMAE were collected at prespecified time points. Patients enrolled at non–PK‐cohort sites had a reduced frequency of PK sample collection. Blood samples for antitherapeutic antibodies (ATAs) were collected predose on Day 1 of cycles 1 to 4, 6, 8, 10, and within 30–37 days after the last study dose. Validated assays were used to measure concentrations of ADC, TAb, and free MMAE in serum or plasma and to assess ATA levels.

### 2.4 Endpoints

The primary efficacy endpoint was confirmed ORR as assessed by IRC. The ORR was defined as the proportion of patients with CR or PR according to RECIST version 1.1. Patients who did not have at least two postbaseline response assessments (initial response and confirmatory scan) were considered nonresponders for analytic purposes. Other primary endpoints included selected PK parameters of ADC, TAb, and free MMAE.

Secondary endpoints were confirmed ORR by investigator assessment; DOR, disease control rate (DCR), and progression‐free survival (PFS) by IRC and investigator assessments; OS; incidence of ATAs to ADC; and safety/tolerability. PFS was defined as the time from the start of the study treatment to the first documentation of objective tumor progression (i.e., progressive disease per RECIST version 1.1) by IRC or investigator assessment (whichever was specified) or to death due to any cause, whichever came first. OS was defined as the time from the start of study treatment to the date of death due to any cause. Based on observed findings and safety data from prior clinical studies of enfortumab vedotin, AEs of special interest with enfortumab vedotin were evaluated, including skin reaction, peripheral neuropathy, and hyperglycemia. AEs of special interest were medical concepts of composite terms based on search criteria (standard Medical Dictionary for Regulatory Activities query or sponsor‐specific query/customized Medical Dictionary for Regulatory Activities query). For example, skin reactions included categories of rash and severe cutaneous adverse reactions (which included specific events such as drug eruption, eczema, dermatitis, mouth ulceration, stomatitis, etc.). Data for treatment‐related AEs (TRAEs) were evaluated by the investigator.

### 2.5 Statistical Methods

The primary efficacy analysis was performed by testing the null hypothesis of the ORR being no more than 10% (historical control rate) against the alternative hypothesis that the ORR is higher than 10% at an overall one‐sided 2.5% level of significance. The study would therefore be considered successful if the lower bound of the two‐sided 95% exact Clopper–Pearson CI for the ORR was higher than 10%. A sample size of 35–40 patients was determined to have sufficient statistical power (95%–97%) to detect a 25% improvement in ORR. As a prespecified exploratory analysis, subgroup analyses were conducted for the primary endpoint based on age, sex, ECOG PS at baseline (0–1), Bellmunt risk score (0–1, ≥ 2), primary tumor site (upper tract, bladder/other), liver metastasis (yes, no), number of prior systemic therapies in a locally advanced or metastatic setting (1–2, ≥ 3), and best response to prior checkpoint inhibitor therapy (responder, nonresponder).

All patients who received any amount of study drug were included in the primary analyses of efficacy endpoints and safety analyses. The PK analyses included data from patients in the PK cohort who received at least 3 of 4 doses of the study drug through cycle 2 Day 1 and had at least five blood samples collected and assayed for measurements of ADC, TAb, or free MMAE concentrations to determine at least one PK parameter.

The ORR 95% CIs were calculated based on the exact Clopper–Pearson method for binomial distribution; Kaplan–Meier estimation was used for time‐to‐event endpoints. Tumor shrinkage was determined as the best percentage change from baseline in the sum of the diameters in the target lesion.

## 3 Results

### 3.1 Study Participants

As of the data cutoff date on May 13, 2022, a total of 40 patients were enrolled at six sites in China (Table 1). The median age was 62 (range, 41–75) years, and 70% (*n* = 28) of patients were younger than 65 years. The majority of patients (*n* = 30 [75.0%]) had an ECOG PS of 1 at baseline. The primary disease site of origin was the upper tract for 29 patients (72.5%). A large majority (*n* = 36 [90.0%]) had UC/transitional cell carcinoma; almost all patients (*n* = 38 [95.0%]) had metastatic disease, including 11 patients (27.5%) with liver metastasis. All patients received prior treatment of immune checkpoint inhibitors and platinum‐based therapy. Almost one‐half (*n* = 18 [45.0%]) of patients had received three or more prior lines of systemic therapy, and 40.0% had received two prior lines of systemic therapy.

**TABLE 1:** Patient demographics and disease characteristics at baseline.

Characteristic	Total (*N* = 40)	PK cohort^a^ (*n* = 15)
Sex		
Male	31 (77.5)	11 (73.3)
Female	9 (22.5)	4 (26.7)
Age, median, year	62	59
Range	41–75	41–75
Race		
Asian	40 (100)	15 (100)
Mean weight (SD), kg	67.6 (12.7)	70.9 (8.6)
Range	42.8–99.0	54.0–90.0
Renal function^b^		
Normal	5 (12.5)	2 (13.3)
Mild impairment	18 (45.0)	8 (53.3)
Moderate impairment	17 (42.5)	5 (33.3)
Bellmunt risk score^c^		
0–1	30 (75.0)	14 (93.3)
≥ 2	10 (25.0)	1 (6.7)
ECOG PS		
0	10 (25.0)	2 (13.3)
1	30 (75.0)	13 (86.7)
Primary disease site^d^		
Upper tract	29 (72.5)	12 (80.0)
Bladder/Other	11 (27.5)	3 (20.0)
Extent of disease^e^		
Metastatic	38 (95.0)	15 (100)
Locally advanced	2 (5.0)	0
Histology at initial diagnosis		
Urothelial carcinoma/Transitional cell carcinoma	36 (90.0)	15 (100)
Urothelial carcinoma/Mixed	4 (10.0)	0
Site of metastasis		
Visceral^f^	31 (77.5)	11 (73.3)
Liver	11 (27.5)	1 (6.7)
Lymph node only	1 (2.5)	0
Prior line of systemic therapy in locally advanced or metastatic setting^g^		
1–2	22 (55.0)	7 (46.7)
≥ 3	18 (45.0)	8 (53.3)
Prior checkpoint inhibitor therapy		
PD‐1 inhibitor^h^	38 (95.0)	15 (100)
PD‐L1 inhibitor^i^	2 (5.0)	0
Prior platinum‐based therapy		
Cisplatin‐based only	23 (57.5)	8 (53.3)
Carboplatin‐based only	10 (25.0)	5 (33.3)
Cisplatin and carboplatin	4 (10.0)	1 (6.7)
Other	3 (7.5)	1 (6.7)
Best overall response to prior checkpoint inhibitor therapy		
Partial response	4 (10.0)	3 (20.0)
Stable disease	4 (10.0)	2 (13.3)
Progressive disease	20 (50.0)	6 (40.0)
Not evaluable	1 (2.5)	1 (6.7)
Unknown	10 (25.0)	2 (13.3)
Not applicable	1 (2.5)	1 (6.7)

*Note:* Values are *n* (%) unless otherwise noted.

Abbreviations: ECOG PS, Eastern Cooperative Oncology Group performance status; NA, not applicable; PD‐1/L1, programmed cell death protein 1/ligand 1; PK, pharmacokinetics.

^a^ Subgroup of total study population.

^b^ Cockcroft–Gault formula used to estimate creatinine clearance (normal, ≥ 90 mL/min; mild impairment, 60 to < 90 mL/min; moderate impairment, 30 to < 60 mL/min).

^c^ The risk factors used to calculate the Bellmunt score include: ECOG PS > 0, presence of liver metastasis, and hemoglobin < 10 g/dL.

^d^ Upper tract included renal pelvis and ureter. Bladder/Other included urethra, bladder, and other.

^e^ Patients with metastatic disease and locally advanced disease were counted as having metastatic disease.

^f^ Patients with baseline tumor results at the lung, liver, spleen, adrenal gland, kidney, heart, colon, bone, or prostate gland.

^g^ Included platinum‐based therapy in the neoadjuvant/adjuvant setting and patient progression ≤ 12 months of therapy completion.

^h^ Tislelizumab, toripalimab, pembrolizumab, camrelizumab, sintilimab, rulonilimab, or nivolumab.

^i^ Durvalumab, atezolizumab, or avelumab.

Fifteen of the 40 patients comprised the PK cohort. Baseline characteristics in the PK cohort were generally similar to those of the overall population (Table 1).

The median duration of treatment was 4.6 (range, 0.6–9.3) months, and median number of cycles completed per patient was 4.0 (range, 1–10). The median relative dose intensity (dose intensity/planned dose intensity × 100) was approximately 74%.

### 3.2 Efficacy

Confirmed ORR by IRC assessment was 37.5% (*n* = 15; 95% CI: 22.7%–54.2%; Table 2). The best overall responses were CR for one patient (2.5%) and PR for 14 patients (35.0%). The median time to response was 1.8 (range, 1.7–3.8) months. Confirmed ORR by investigator assessment was 42.5% (*n* = 17; 95% CI: 27.0%–59.1%).

**TABLE 2:** Summary of treatment response.

Response	IRC (*N* = 40)	Investigator (*N* = 40)
Confirmed objective response rate (95% CI^a^), %	37.5 (22.7–54.2)^b^	42.5 (27.0–59.1)
Confirmed best overall response,^c^ *n* (%)		
CR	1 (2.5)	0
PR	14 (35.0)	17 (42.5)
Stable disease^d^	14 (35.0)	16 (40.0)
Progressive disease	7 (17.5)	4 (10.0)
NE^e^	4 (10.0)	3 (7.5)
Confirmed disease control rate^f^ (95% CI^a^), %	72.5 (56.1–85.4)	82.5 (67.2–92.7)
Duration of response,^g^ months		
Median (95% CI)	NE (2.04–NE)	NE (2.04–NE)
Range	1.84+, 7.49+	1.64+, 7.49+
Rate of duration of response (95% CI),^h^, ^i^ %		
At 6 months	59.7 (22.2–83.8)	61.1 (26.2–83.4)
At 12 months	NE (NE–NE)	NE (NE–NE)
Overall survival at 6 months (95% CI),^h^ %	78.0 (60.1–88.6)	
Median (95% CI) follow‐up of overall survival, months	6.5 (5.3–6.8)	

*Note:* Censoring is indicated by the + symbol.

Abbreviations: CR, complete response; IRC, independent review committee; NE, not evaluable; PR, partial response; RECIST, Response Evaluation Criteria in Solid Tumors.

^a^ Two‐sided 95% CIs based on exact (Clopper–Pearson) binomial distribution.

^b^ Primary efficacy endpoint.

^c^ Defined by RECIST v1.1. CR and PR confirmed by two scans ≥ 4 week apart.

^d^ Minimum duration for stable disease was 49 days.

^e^ Includes patients with no postbaseline tumor assessment data.

^f^ Defined by patients with CR, PR, or stable disease according to RECIST v1.1.

^g^ Duration of response based on Kaplan–Meier estimate; CI based on log–log transform. CR or PR.

^h^ Rate and 95% CI estimated using Kaplan–Meier method and Greenwood formula.

^i^ Indicates the probability of remaining in response after 6 or 12 months (as indicated) from their first documented response.

The DCRs were 72.5% (*n* = 29; 95% CI: 56.1%–85.4%) by IRC and 82.5% (*n* = 33; 95% CI: 67.2%–92.7%) by investigator assessment. Median DOR by IRC or investigator assessment was not reached because 11 of 15 responders by IRC and 13 of 17 responders by investigator assessment had ongoing responses at the data cutoff date.

Subgroup analysis of ORR by select demographics and disease characteristics (Figure S1) demonstrated that the lower bound of the 95% CI was higher than 10% (historical benchmark) for most subgroups, including patients with upper tract UC, consistent with the findings for the overall population. Of note, the ORR was numerically higher among patients with primary tumor sites in bladder or “other” than in the upper tract (63.6% [n/*N* = 7/11] vs. 27.6% [n/*N* = 8/29]) and was higher among patients with a baseline ECOG PS of 0 than in those with a baseline ECOG PS of 1 (70.0% [n/*N* = 7/10] vs. 26.7% [n/*N* = 8/30]). In addition, responses were observed in patients with liver metastases (45.5% [n/*N* = 5/11]) and in those who had received three or more lines of systemic therapy (33.3% [n/*N* = 6/18]).

**FIGURE 1:**
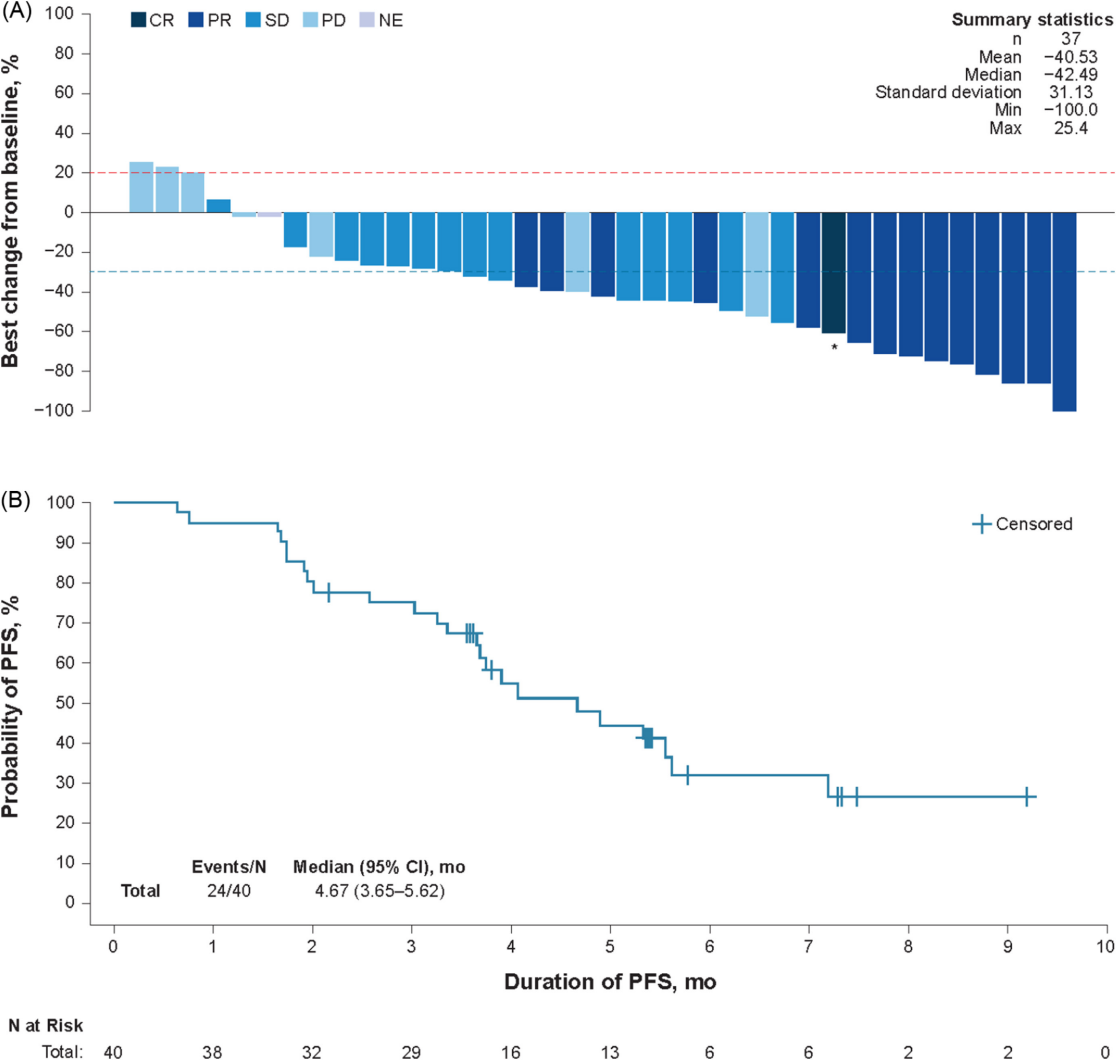
Antitumor effects of enfortumab vedotin in patients with locally advanced or metastatic urothelial carcinoma. (A) Best percentage change from baseline in size of target lesions (by IRC), and (B) progression‐free survival (IRC assessed). In panel A, dashed lines reflect RECIST version 1.1 percentage change thresholds for PD (red; 20% increase) and PR (blue; 30% reduction) [42]. *The only target lesion for the patient with CR was a lymph node. Abbreviations: CR, complete response; IRC, independent review committee; NE, not evaluable; PD, progressive disease; PR, partial response; RECIST, Response Evaluation Criteria in Solid Tumors; SD, stable disease.

Among 37 patients with baseline and postbaseline tumor assessments, target lesions had a reduction in the sum of diameters from baseline by IRC assessment in 33 patients (89.2%), and 24 patients (64.9%) had reductions of 30% or more from baseline (Figure 1A). Median PFS was 4.7 and 4.2 months by IRC and investigator assessments, respectively (Figure 1B). At a median follow‐up time of 6.5 months, the median OS was not reached (lower bound of 95% CI: 6.74) (Figure S2). The OS rate at 6 months was 78.0% (95% CI: 60.1%–88.6%).

### 3.3 Pharmacokinetics

The final PK analysis population included 13 patients. A summary of PK parameters for ADC, TAb, and free MMAE on days 1 and 15 of cycle 1 is provided in Table 3. Figure S3A–C presents mean serum concentration–time profiles for ADC and TAb and mean plasma‐free MMAE in the PK cohort during cycle 1. Serum concentrations of ADC and TAb reached peaks at the end of the enfortumab vedotin infusion. By contrast to ADC and TAb values, free MMAE concentrations continued to increase after the end of the infusion, peaking approximately 2–3 days postinfusion. Minimal ADC, TAb, and free MMAE accumulation was apparent with repeat dosing of enfortumab vedotin as suggested by mean accumulation ratios (third to first dose) for maximum concentration and area under the concentration‐time curve from time 0 to 7 days ranging from 1.03 to 1.32. A summary of concentrations from the PK and non‐PK cohorts is shown in Table S1.

**TABLE 3:** PK parameters for ADC, TAb, and free MMAE in the PK cohort.^a^

Parameter	Cycle 1 Day 1	Cycle 1 Day 15
ADC, *n*	13^b^	10
AUC_0–7d_, day μg/mL	32.4 (6.2)	36.5 (7.9)
*C*_max_, μg/mL	28.1 (4.52)	27.6 (4.01)
*R*_ac_ (*C*_max_)	NA	1.03 (0.15)
*R*_ac_ (AUC_0–7d_)	NA	1.20 (0.228)
TAb, *n*	13^b^	10
AUC_0–7d_, day μg/mL	77.4 (17.3)	94.7 (15.7)
*C*_max_, μg/mL	31.7 (5.73)	34.4 (4.64)
*R*_ac_ (*C*_max_)	NA	1.13 (0.137)
*R*_ac_ (AUC_0–7d_)	NA	1.29 (0.208)
Free MMAE, *n*	13^b^	10
AUC_0–7d_, day ng/mL	16.9 (9.2)	23.3 (16.4)
*C*_max_, ng/mL	3.3 (1.74)	4.5 (3.58)
Median *t*_max_ (range), days	2.00 (1.87–2.93)	1.93 (0.86–2.92)
*R*_ac_ (C_max_)	NA	1.28 (0.499)
*R*_ac_ (AUC_0–7d_)	NA	1.32 (0.486)

*Note:* Data shown as mean (SD) unless noted otherwise.

Abbreviations: ADC, antibody–drug conjugate; AUC_0–7d_, area under the concentration‐time curve from time 0–7 days; *C*_max_, maximum concentration; MMAE, monomethyl auristatin E; NA, not applicable; *R*_ac_ (AUC_0–7d_), accumulation ratio calculated using AUC_0–7d_; *R*_ac_ (*C*_max_), accumulation ratio calculated using *C*_max_; TAb, total antibody; *t*_max_, time of maximum concentration.

^a^ Data reflect participants who received ≥ 3 of 4 doses through cycle 2 day 1 and had ≥ 5 blood samples collected and assayed for measurement of ADC, TAb, and free MMAE serum/plasma concentrations to determine ≥ 1 PK parameter. Range of N values for each PK parameter, 10–13.

^b^ Data available for 13 patients for *C*_max_; data available for 12 patients for AUC_0–7d_.

### 3.4 Immunogenicity

A total of 38 participants were tested for ATAs against enfortumab vedotin. As of the ATA sample collection cutoff date (March 18, 2022), the incidence of positive ATAs postbaseline was 0%.

### 3.5 Safety

All 40 patients experienced at least one TRAE of any grade (Table 4). The most common TRAEs (≥ 40% of patients) were anemia, increased aspartate aminotransferase level, decreased appetite, nausea, diarrhea, hyperglycemia, decreased neutrophil count, increased alanine aminotransferase level, rash, constipation, and decreased white blood cell count. Grade 3 or higher TRAEs were reported in 29 patients (72.5%), the most common (> 10% of patients) being decreased neutrophil count, anemia, rash, decreased white blood cell count, and hyponatremia. A summary of any grade treatment‐emergent AEs reported in ≥ 30% of patients or grade ≥ 3 treatment‐emergent AEs in ≥ 10% of patients is presented in Table S2.

**TABLE 4:** Treatment‐related adverse events of any grade (≥ 30%) or grade ≥ 3 (≥ 10%) by MedDRA preferred term.

Adverse event	Any grade (*N* = 40)	Grade ≥ 3 (*N* = 40)
Any	40 (100)	29 (72.5)
Anemia	27 (67.5)	6 (15.0)
Aspartate aminotransferase increased	25 (62.5)	1 (2.5)
Decreased appetite	25 (62.5)	1 (2.5)
Nausea	20 (50.0)	0
Neutrophil count decreased	19 (47.5)	8 (20.0)
Hyperglycemia	19 (47.5)	3 (7.5)
Diarrhea	19 (47.5)	2 (5.0)
Rash	17 (42.5)	5 (12.5)
Alanine aminotransferase increased	17 (42.5)	1 (2.5)
White blood cell count decreased	16 (40.0)	5 (12.5)
Constipation	16 (40.0)	0
Hypokalemia	14 (35.0)	2 (5.0)
Pruritus	14 (35.0)	2 (5.0)
Hyponatremia	12 (30.0)	5 (12.5)
Alopecia	12 (30.0)	0
Hypophosphatemia	7 (17.5)	4 (10.0)
Rash maculopapular	6 (15.0)	4 (10.0)

*Note:* Values are *n* (%). *N* = 40.

Abbreviation: MedDRA, Medical Dictionary for Regulatory Activities.

Serious TRAEs were reported in 16 patients (40.0%), the most common (≥ 5% of patients) being pneumonia, rash, diabetes mellitus, drug eruption, leukopenia, and decreased neutrophil count. Dose reduction due to TRAEs occurred in 25 patients (62.5%), most commonly due to rash (22.5%). Dose interruption due to TRAEs occurred in 22 patients (55.0%), most commonly due to fatigue (10.0%). Treatment discontinuation due to TRAEs occurred in two patients (5.0%) (acute coronary syndrome, *n* = 1; hyperglycemia and rash, *n* = 1). One patient (2.5%) died due to a TRAE of acute coronary syndrome. Two patients (5.0%) died following malignant neoplasm progression that was not considered treatment‐related.

Skin reactions, hyperglycemia, and peripheral neuropathy were the most frequent TRAEs of special interest with enfortumab vedotin (Table 5). Treatment‐related skin reactions were reported in 31 patients (77.5%); grade 3 reactions occurred in 13 patients (32.5%) and all others were grade 1 or 2. One patient discontinued treatment due to a serious grade 3 rash that required hospitalization. Most skin reactions resolved after dose interruption or reduction and supportive care measures, and patients were able to continue study treatment. Treatment‐related hyperglycemia was reported in 22 patients (55.0%); the majority of events were grade 1 or 2. Grade 3 and 4 events were reported in four patients (10.0%) and one patient (2.5%), respectively; all patients recovered or were recovering after drug interruption or supportive care. Hyperglycemia led to treatment discontinuation in one patient (2.5%). Treatment‐related peripheral neuropathy was reported in 15 patients (37.5%), and all events were grade 1 or 2. No events led to treatment discontinuation.

**TABLE 5:** Treatment‐related adverse events of special interest^a^ with enfortumab vedotin of any grade and grade ≥ 3.

Adverse event	Any grade (*N* = 40)	Grade ≥ 3 (*N* = 40)
Skin reaction^b^	31 (77.5)	13 (32.5)
Hyperglycemia^c^	22 (55.0)	5 (12.5)
Peripheral neuropathy^d^	15 (37.5)	0
Dry eye	3 (7.5)	0
Corneal disorder/blurred vision	2 (5.0)	0
Infusion‐related reaction	2 (5.0)	0

*Note:* Values are *n* (%). *N* = 40.

Abbreviations: CMQ, Customized Medical Query; MedDRA, Medical Dictionary for Regulatory Activities; SMQ, Standard MedDRA Query; SSQ, Sponsor‐Specific Query.

^a^ Composite of related preferred terms identified by standard Medical Dictionary for Regulatory Activities query or sponsor‐specified query.

^b^ Composite term of rash (SSQ/CMQ), including rash, rash maculo‐papular, drug eruption, eczema, erythema, dermatitis, dermatitis contact, and rash macular, and severe cutaneous adverse reactions (SMQ), including mouth ulceration, skin exfoliation, drug eruption, stomatitis, and dermatitis exfoliative.

^c^ Composite term of hyperglycemia (SSQ/CMQ), including hyperglycemia, increased blood glucose, diabetes mellitus, and increased glycosylated hemoglobin.

^d^ Composite term of peripheral neuropathy motor events (SSQ/CMQ), including muscular weakness, and peripheral neuropathy sensory events (SSQ/CMQ), including neuropathy peripheral, hypoesthesia, peripheral sensory neuropathy, and neuralgia.

## 4 Discussion

In this single‐arm, open‐label, bridging study, treatment with enfortumab vedotin resulted in clinically meaningful efficacy in Chinese patients with locally advanced or mUC who previously received platinum‐based chemotherapy and PD‐1/L1 inhibitor therapy. Efficacy findings were consistent with those from the phase 2 EV‐201 trial and phase 3 EV‐301 trial of enfortumab vedotin in larger and globally diverse populations [27, 29]. Notably, 94% of the patients were from the United States in cohort 1 of EV‐201, and the geographical distribution of EV‐301 was 42% from Western Europe, 14% from the United States, and 44% from the rest of the world. The confirmed ORR by IRC in this Chinese population was 37.5% (95% CI: 22.7%–54.2%) versus 44% (95% CI: 35.1%–53.2%) in cohort 1 of EV‐201 (*n* = 125) [27], and the estimated median PFS was 4.7 months compared with 5.6 months in EV‐301 [29]. The probability of OS at 6 months was 78% in the present study and approximately 80% in EV‐301 [29]. It is worth noting that the Chinese patients enrolled in the present study had received more lines of previous anticancer therapy than patients in EV‐301. In the present study, 45% of patients had three or more prior lines of systemic therapy versus 13% of patients in EV‐301 [29]. The proportion of patients with upper tract disease (72.5%) was higher in the current study and in the Japanese subgroup analysis of EV‐301 (61.1%) [31] than in the overall population of EV‐301 (32.6%) [29]; however, this is in line with the higher incidence of upper tract UC previously noted in Asian populations [32, 33], as well as proportions reported in recent trials of Chinese patients with locally advanced or mUC in the last 5 years [34, 35, 36, 37]. In EV‐301, subgroup analysis by primary tumor site showed no difference in OS between subgroups with upper urinary tract tumors versus those with bladder/other site tumors (HR 0.85 [95% CI: 0.57–1.27] |vs. 0.67 [95% CI: 0.51–0.88], respectively) [29]; similarly, ORRs for these subgroups in the current study had overlapping 95% CIs.

The type and incidence of TRAEs in this population of Chinese patients, including TRAEs of special interest, were consistent with the known safety profile of enfortumab vedotin, and no new safety signals were identified. Two patients discontinued enfortumab for TRAEs, whereas almost two‐thirds of patients (62.5%) had a reduction in dose in response to a TRAE. This indicates that most TRAEs could be managed with dose modification, thereby allowing patients who were receiving benefits to continue treatment.

PK parameters of ADC, TAb, and free MMAE were well characterized in this Chinese study population and appeared generally consistent with previously characterized parameters obtained from a North American, predominantly White, study population [38]. Serum concentrations of ADC and TAb reached their peak at the end of the enfortumab vedotin infusion. By contrast, MMAE concentrations continued to increase after the infusion, with median peak MMAE concentrations attained approximately 2 days later. Minimal ADC, TAb, and MMAE accumulation rates were observed after repeated enfortumab vedotin dosing. No treatment‐emergent ATAs against enfortumab vedotin were observed. PK parameters in patients in China in this study were consistent with that of the global population; based on these PK data, ethnicity has no clinically meaningful effect on exposures of ADC and free MMAE [39].

Limitations for consideration in this study included the nonrandomized, single‐arm design and small sample size. Additionally, the median follow‐up time of 6.5 months may prevent the determination of a median DOR or median OS; however, although the follow‐up is short for OS, a final analysis is planned.

Treatment options beyond second‐line therapy for locally advanced or mUC have been evaluated in clinical trials [21, 29]; however, no drug has been approved in patients with advanced UC who previously received platinum‐based chemotherapy and PD‐1/L1 inhibitor therapy in China [13]. Recently studied agents for Chinese patients with advanced UC include the humanized monoclonal antibody against PD‐1 tislelizumab, the humanized immunoglobulin G4 monoclonal antibody against human PD‐1 toripalimab, and the HER2‐targeting ADC disitamab vedotin [34, 36, 40], all of which have received approval from the National Medical Products Administration in China as second‐line options. Pooled data from two phase 2 studies of disitamab vedotin in patients with histologically HER2‐positive (defined as IHC2+ or 3+), unresectable locally advanced or mUC whose disease had progressed on at least one line of systemic chemotherapy demonstrated an ORR of 50.5% (95% CI: 40.6%–60.3%) [41]. The disitamab vedotin phase two trials were single‐arm studies with small sample sizes (combined *N* = 107) and enrolled only patients with HER2‐positive disease. Furthermore, only 25% of the study sample had received prior PD‐1/L1 treatment [41], as opposed to 100% of patients in the present study. Clinically relevant differences in study‐sample characteristics between these trials and the current trial preclude meaningful between‐trial comparisons, and further studies will be necessary to clarify best practices for treatment selection based on prior treatment with PD‐1/L1 inhibitors and HER2‐expression status.

## 5 Conclusions

Treatment with enfortumab vedotin demonstrated meaningful clinical activity and a manageable safety profile in Chinese patients with locally advanced or mUC previously treated with platinum‐based chemotherapy and PD‐1/L1 inhibitor therapy, a disease with limited treatment options. The efficacy, safety, and PK results in Chinese patients were generally consistent with those observed in the global population.

## Author Contributions

**Siming Li:** conceptualization (equal), investigation (equal), methodology (equal), resources (equal), writing – original draft (equal), writing – review and editing (equal). **Yanxia Shi:** investigation (equal), resources (equal), writing – original draft (equal), writing – review and editing (equal). **Haiying Dong:** investigation (equal), resources (equal), writing – original draft (equal), writing – review and editing (equal). **Hongqian Guo:** investigation (equal), resources (equal), writing – original draft (equal), writing – review and editing (equal). **Yu Xie:** investigation (equal), resources (equal), writing – original draft (equal), writing – review and editing (equal). **Zhongquan Sun:** investigation (equal), resources (equal), writing – original draft (equal), writing – review and editing (equal). **Xiaoping Zhang:** investigation (equal), resources (equal), writing – original draft (equal), writing – review and editing (equal). **Eric Kim:** writing – original draft (equal), writing – review and editing (equal). **Jun Zhang:** formal analysis (equal), writing – original draft (equal), writing – review and editing (equal). **Yue Li:** conceptualization (equal), data curation (equal), formal analysis (equal), methodology (equal), writing – original draft (equal), writing – review and editing (equal). **Chenming Xu:** conceptualization (equal), data curation (equal), formal analysis (equal), methodology (equal), writing – original draft (equal), writing – review and editing (equal). **Haishan Kadeerbai:** formal analysis (equal), writing – original draft (equal), writing – review and editing (equal). **Sue Lee:** writing – original draft (equal), writing – review and editing (equal). **Seema Gorla:** formal analysis (equal), writing – original draft (equal), writing – review and editing (equal). **Jun Guo:** conceptualization (equal), investigation (equal), methodology (equal), resources (equal), writing – original draft (equal), writing – review and editing (equal). **Xinan Sheng:** conceptualization (equal), investigation (equal), methodology (equal), resources (equal), writing – original draft (equal), writing – review and editing (equal).

## Ethics Statement

The protocol and amendments were approved by site‐independent review boards or ethics committees. The study was conducted in accordance with the Declaration of Helsinki, the Council for International Organizations of Medical Sciences ethical guidelines, and applicable International Council for Harmonisation of Technical Requirements for Pharmaceuticals for Human Use Good Clinical Practice guidelines. All patients (or a legally authorized representative) provided written informed consent.

## Conflicts of Interest

Siming Li, Yanxia Shi, Haiying Dong, Hongqian Guo, Yu Xie, Zhongquan Sun, and Xiaoping Zhang have no disclosures to report. Eric Kim is an employee of Pfizer Inc. Jun Zhang is an employee of Astellas Pharma Inc. Yue Li is an employee of Astellas Pharma Inc. Chenming Xu is an employee of Astellas Pharma Inc. Haishan Kadeerbai is an employee of Astellas Pharma Inc. Sue Lee was an employee of Astellas Pharma Inc. at the time of the study conduct and manuscript initiation. Seema Gorla is an employee of Astellas Pharma Inc. Jun Guo serves in consulting/advisory roles in Merck Sharp & Dohme, Roche, Bayer, Novartis, Simcere Pharmaceutical Group, Shanghai Junshi Biosciences, and Oriengene. Xinan Sheng reports a non‐profit consulting or advisory role in Pfizer, Astellas, AstraZeneca, MSD, Novartis, RemeGen, and Junshi Biosciences.

## Supporting information


Data S1.

## Data Availability

Researchers may request access to anonymized participant level data, trial level data, and protocols from Astellas Pharma Inc.–sponsored clinical trials at www.clinicalstudydatarequest.com. For the Astellas Pharma Inc. criteria on data sharing see: https://clinicalstudydatarequest.com/Study‐Sponsors/Study‐Sponsors‐Astellas.aspx.
