# Supplementary material for: Phase 2 Trial of Enfortumab Vedotin in Patients With Previously Treated Locally Advanced or Metastatic Urothelial Carcinoma in China
Source: Cancer Med. 2024 Nov 12;13(21):e70368. doi: 10.1002/cam4.70368 (PMC11555717; doi:10.1002/cam4.70368)

**SUPPORTING INFORMATION**

**Supplemental Table S1.** Summary serum or plasma concentrations of ADC, TAb, and free MMAE in the PK and non-PK cohorts at end of infusion for cycle 1

| **Parameter** | **PK cohort** | **Non-PK cohort** |
| --- | --- | --- |
| ADC,^†^ ng/mL [n] |  |  |
| Day 1 | 27,700 (4410) [13] | 25,300 (3830) [23] |
| Day 8 | 27,400 (4390) [12] | 24,900 (5400) [17] |
| Day 15 | 27,500 (3820) [10] | 22,000 (5980) [11] |
| TAb,^‡^ ng/mL [n] |  |  |
| Day 1 | 30,800 (5730) [13] | 29,100 (5050) [23] |
| Day 8 | 34,300 (6640) [12] | 29,900 (5450) [17] |
| Day 15 | 33,600 (5450) [10] | 27,600 (6210) [11] |
| Free MMAE,^§^ pg/mL [n] |  |  |
| Day 1 | 357 (154) [13] | 376 (241) [23] |
| Day 8 | 1680 (1290) [12] | 2720 (1490) [17] |
| Day 15 | 2110 (1680) [10] | 3350 (2600) [11] |

Data shown as mean (SD) unless noted otherwise. Select data shown; serum and plasma concentrations for ADC, TAb, and free MMAE were also collected at other prespecified sampling times.

ADC, antibody–drug conjugate; MMAE, monomethyl auristatin E; TAb, total antibody.

^†^Concentrations below the lower limit of quantification of 23.6 ng/mL were set to 0.

^‡^Concentrations below the lower limit of quantification of 25.0 ng/mL were set to 0.

^§^Concentrations below the lower limit of quantification of 10.0 pg/mL were set to 0.

**Supplemental Table S2.** Treatment-emergent adverse events of any grade (≥30%) or grade ≥3 (≥10%) by MedDRA preferred term

| **Adverse event** | **Any grade**  **(N=40)** | **Grade ≥3**  **(N=40)** |
| --- | --- | --- |
| Any | 40 (100) | 30 (75.0) |
| Anemia | 28 (70.0) | 6 (15.0) |
| Aspartate aminotransferase increased | 26 (65.0) | 1 (2.5) |
| Decreased appetite | 26 (65.0) | 1 (2.5) |
| Hyperglycemia | 20 (50.0) | 3 (7.5) |
| Nausea | 20 (50.0) | 0 |
| Constipation | 19 (47.5) | 0 |
| Neutrophil count decreased | 19 (47.5) | 8 (20.0) |
| Diarrhea | 19 (47.5) | 2 (5.0) |
| Alanine aminotransferase increased | 18 (45.0) | 1 (2.5) |
| Rash | 17 (42.5) | 5 (12.5) |
| White blood cell count decreased | 16 (40.0) | 5 (12.5) |
| Hypokalemia | 15 (37.5) | 2 (5.0) |
| Pyrexia | 15 (37.5) | 0 |
| Pruritus | 14 (35.0) | 2 (5.0) |
| Hyponatremia | 13 (32.5) | 5 (12.5) |
| Alopecia | 12 (30.0) | 0 |
| Vomiting | 12 (30.0) | 0 |
| Hypophosphatemia | 7 (17.5) | 5 (12.5) |
| Rash maculo-papular | 6 (15.0) | 4 (10.0) |
| Pneumonia | 5 (12.5) | 5 (12.5) |

Values are n (%).

Abbreviation: MedDRA, Medical Dictionary for Regulatory Activities.

**Supplemental Figure S1**. Forest plot for subgroup analysis of confirmed ORR per independent review committee. In the forest plot, the vertical dashed line indicates a historical benchmark of 10%. Abbreviations: ECOG PS, Eastern Cooperative Oncology Group performance status; ORR, objective response rate.


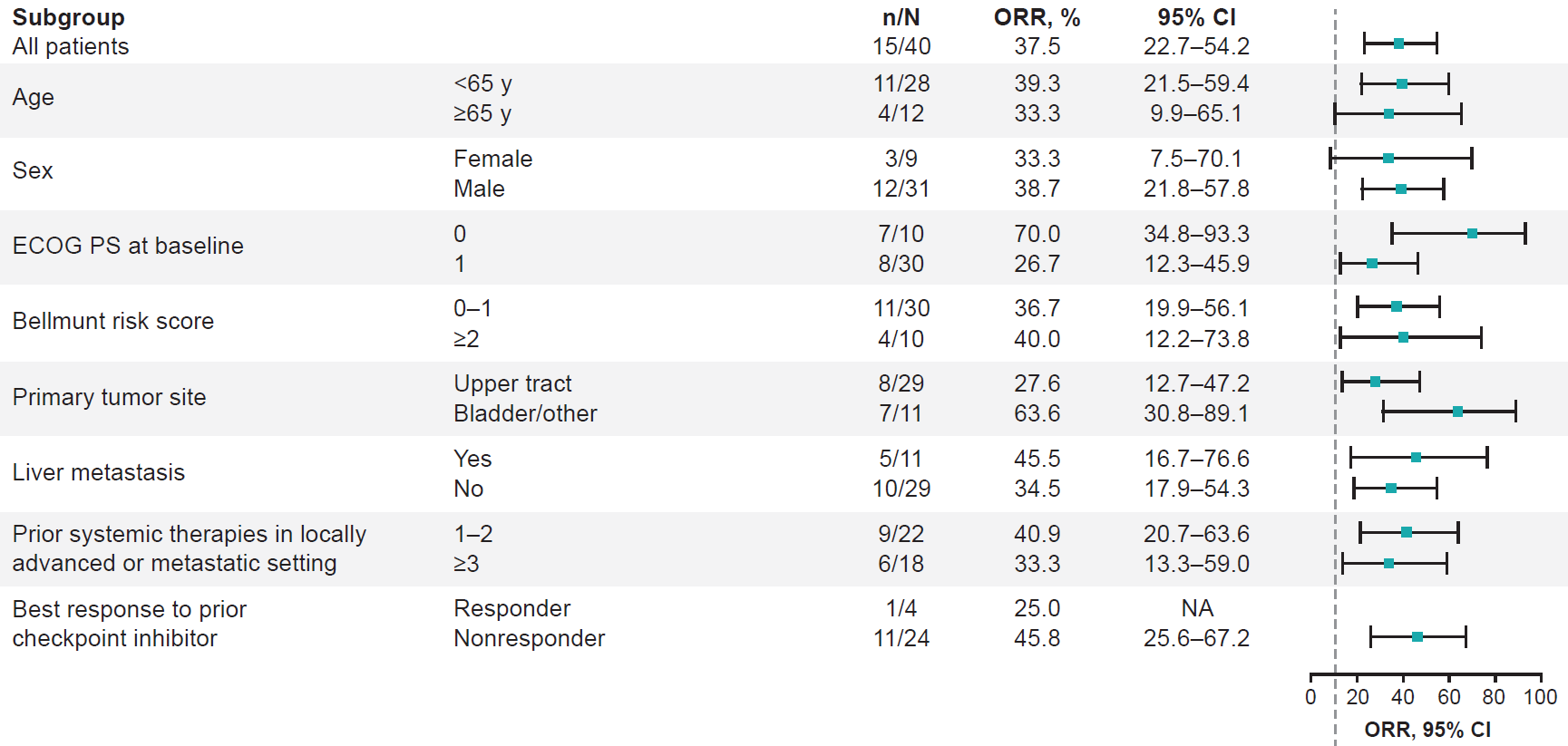


**Supplemental Figure S2**. Overall survival. Abbreviations: NE, not evaluable; OS, overall survival.

**
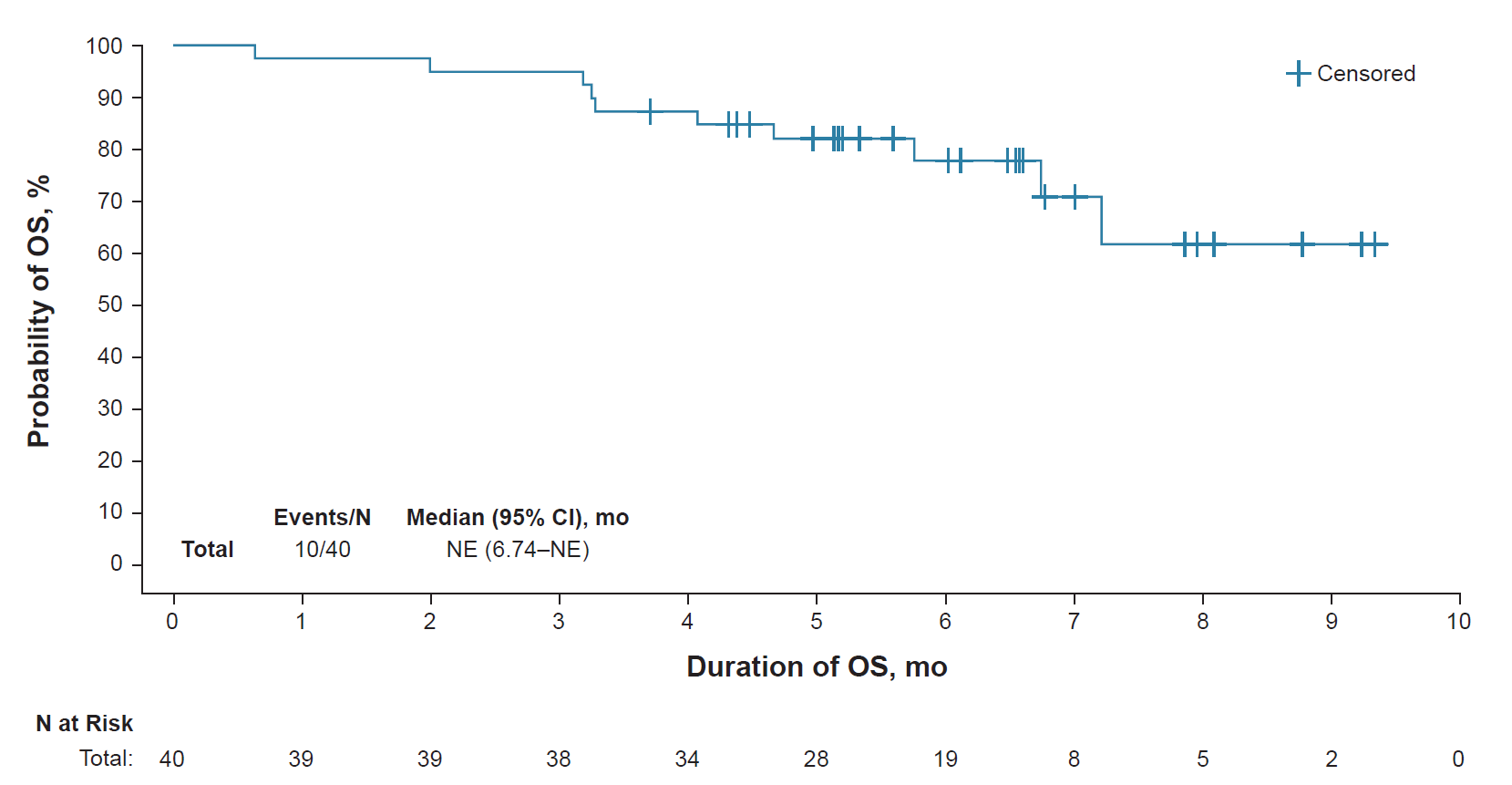
**

**Supplemental Figure S3**. Mean serum concentration profiles of (A) antibody–drug conjugate (semilog scale plot), (B) total antibody (semilog scale plot), and (C) mean (SD) plasma concentration profile (semilog scale plot) of free monomethyl auristatin E in the PK cohort (n=13) during cycle 1 of enfortumab vedotin 1.25 mg/kg on days 1, 8, and 15. Abbreviation: LLOQ, lower limit of quantitation.

(A)


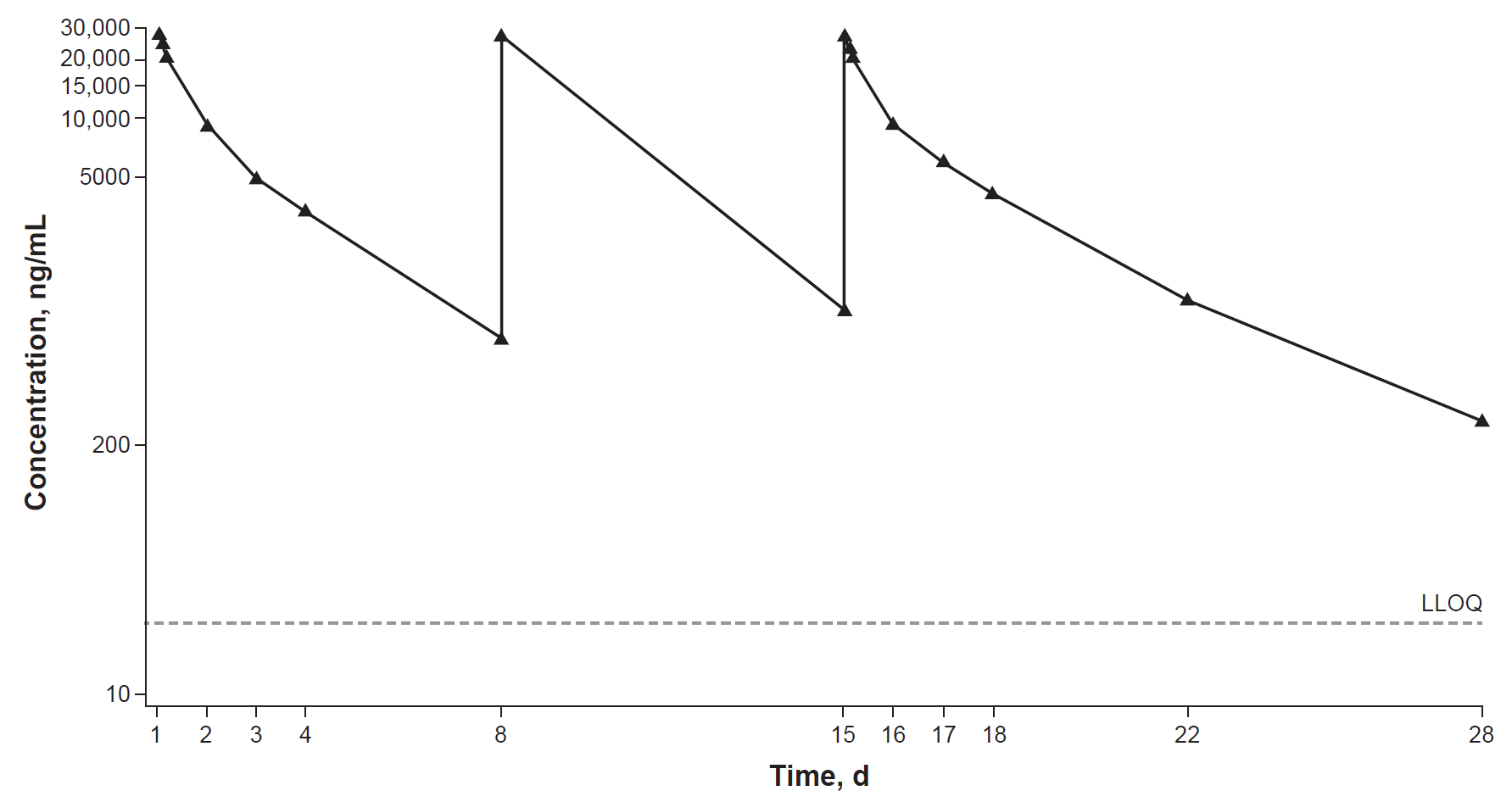


(B)


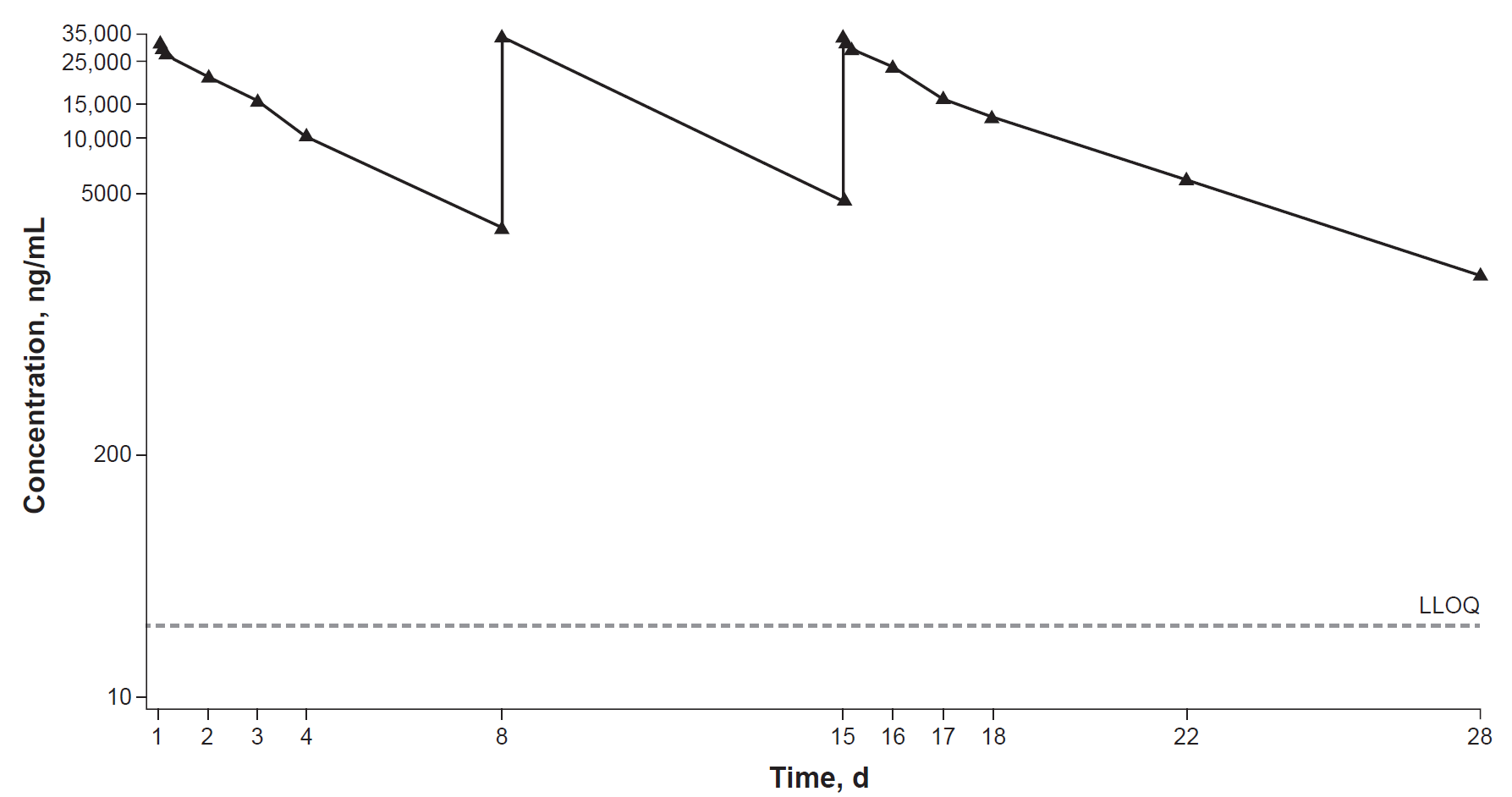


(C)


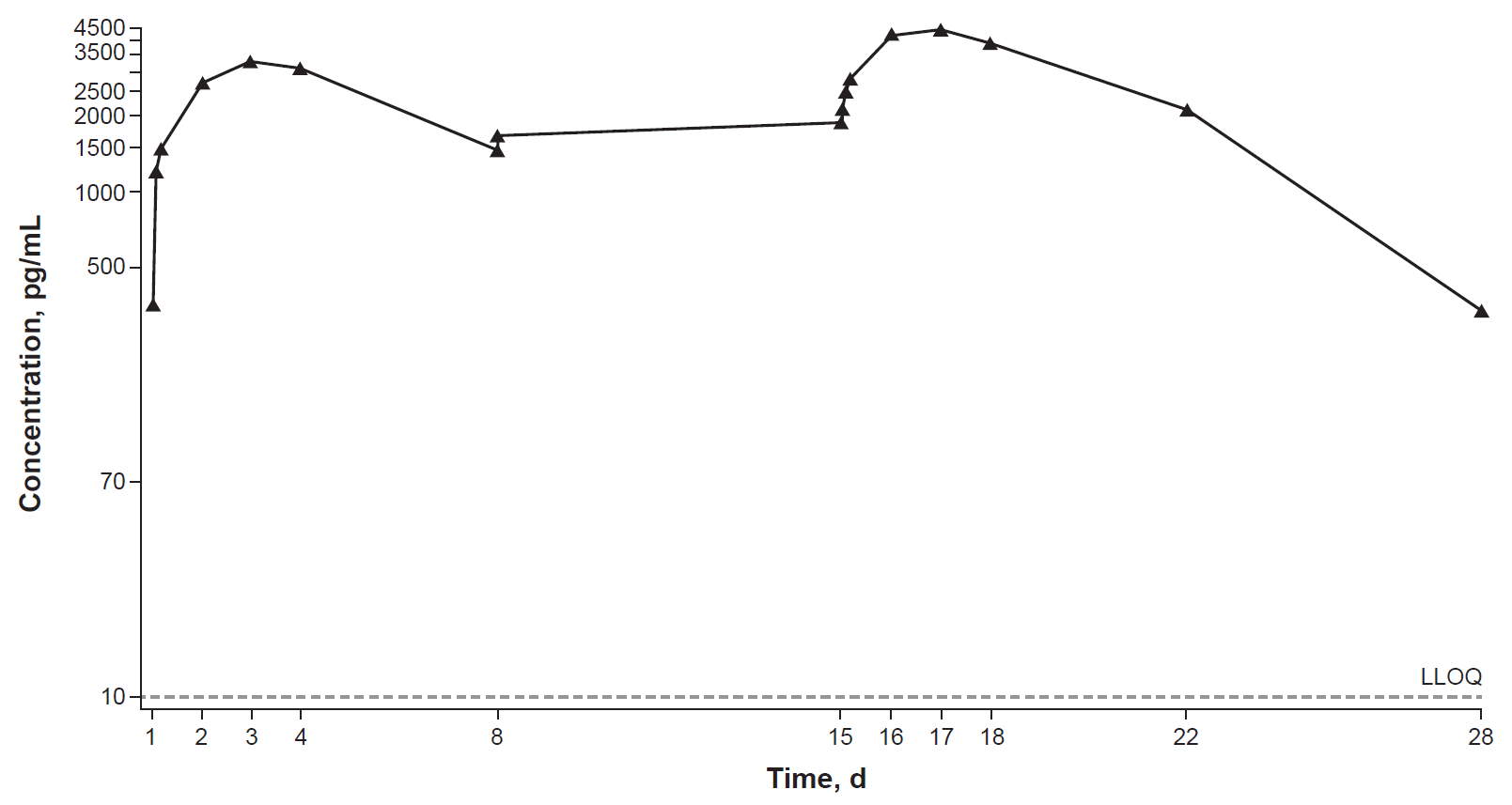

Supplement: Supplementary file 1 — Data S1. [file CAM4-13-e70368-s001.docx]
